# Oleosome interfacial engineering to enhance their functionality in foods

**DOI:** 10.1016/j.crfs.2024.100682

**Published:** 2024-01-17

**Authors:** Saeed M. Ghazani, Jason Hargreaves, Burcu Guldiken, Analucia Mata, Erica Pensini, Alejandro G. Marangoni

**Affiliations:** a Department of Food Science, University of Guelph, Guelph, Ontario, Canada; b Botaneco, Calgary, Alberta, Canada; c College of Engineering and Physical Sciences, University of Guelph, Guelph, Ontario, Canada

**Keywords:** Oleosome, Stability, Xanthan, Gellan, Phospholipid, Glycerol

## Abstract

This study aimed to increase the physical stability of native sunflower oleosomes to expand their range of applications in food. The first objective was to increase the stability and functionality of oleosomes to lower pH since most food products require a pH of 5.5 or lower for microbial stability. Native sunflower oleosomes had a pI of 6.2. One particularly effective strategy for long-term stabilization, both physical and microbial, was the addition of 40% (w/w) glycerol to the oleosomes plus homogenization, which decreased the pI to 5.3 as well as decreasing oleosome size, narrowing the size distribution and increasing colloidal stability. Interfacial engineering of oleosomes by coating them with lecithin and the polysaccharides xanthan and gellan, effectively increased stability, and lowered their pI to 3.0 for lecithin and lower than 3.0 for xanthan. Coating oleosomes also caused a greater absolute value of the ζ-potential; for example, this amount was shifted to −20 mV at pH 4.0 for xanthan and to −28 mV at pH 4.0 for lecithin, which provides electrostatic stabilization. Polysaccharides also provide steric stabilization, which is superior. A significant increase in the diameter of coated oleosomes was observed with lecithin, xanthan and gellan. The oleosome sample with 40% glycerol showed high storage stability at 4 °C (over three months). The addition of glycerol also decreased the water activity of the oleosome suspension to 0.85, which could prevent microbial growth.

## Highlights

• Native oleosomes display low colloidal stability when the pH is lowered below their isoelectric point of ∼6.2
• Coating oleosomes with lecithin, xanthan or gellan increased their stability at low pH.
• Coated oleosomes had a lower isoelectric point and a ζ-potential.
• Oleosome suspension containing 40% (w/w) glycerol were very stable upon storage at 4 °C, at a lower water activity of 0.85

## Graphical abstract

**Figure:**
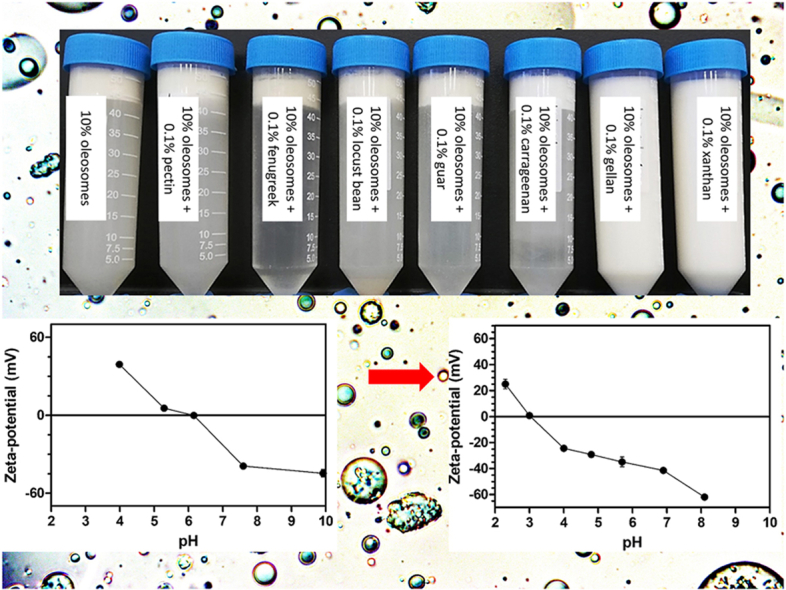

## 1 Introduction

In higher plants, oleosomes are naturally emulsified oil droplets containing triacylglycerols (TAGs) in the core (94–98% w/w), covered and stabilized by a unique protein/phospholipid membrane layer. This unique structure makes them form a natural oil-in-water suspension which serves as energy stores for the germination and growth of seedlings (Guzha et al., 2023; Tzen and Huang, 1992). Isolated oleosomes from different sources have a spherical shape and possess diameters ranging from 0.2 to 2.5 μm controlled by the relative TAG to oleosin (structural proteins found on oleosome surface) ratio (Nikiforidis, 2019). However, previous studies showed that the oleosome extraction method greatly affects their particle size, ranging from nanoscale to microscale (Nikiforidis et al., 2013). The oleosome membranes consist mostly of phosphatidylcholine (PC) and phosphatidylserine (PS) monolayers (about 2% w/w of oleosome), that are negatively charged and interact through electrostatic attractive forces with the basic amino acid residues of surface proteins (oleosins, caleosins, and steroleosins), whose content is about 0.6–3.0% w/w (Guzha et al., 2023; Tzen et al., 1993; Shimada and Hara-Nishimura, 2010; Deleu et al., 2010). Among surface proteins, oleosins are the main proteins which are small alkaline proteins with a molecular mass ranging from 15 to 26 kDa (Tzen and Huang, 1992). Amino acid sequence analysis of oleosins has shown the existence of three structural domains, including an amphipathic N-terminal domain, a central hydrophobic hairpin structure domain that is pinned in the TAG core, and an amphipathic α-helical domain near the C-terminus. These secondary structures cause the protein to reside stably on the phospholipid monolayer, at the surface of the oleosomes (Jolivet et al., 2004). While caleosins have a shorter hydrophobic domain similar to oleosins (Frandsen et al., 2001), they contain an N-terminal region that is hydrophilic with a single Ca^2+^ binding site exposed to the aqueous phase (Frandsen et al., 1996). Moreover, the aqueous phase's polarity significantly influences the secondary structure of caleosins, and their properties might change in different environments (Purkrtova et al., 2007). Steroleosins are relatively bigger proteins (above 35 kDa) with a hydrophobic anchoring segment of similar size as caleosins and a hydrophilic domain in contact with the aqueous phase (Lin et al., 2002).

Oleosomes are natural emulsions and can be used as emulsifiers, and thus could have many applications in foods, such as sauces, salad dressing, coffee whitener, mayonnaise, milk cream alternatives, yogurt, and ice cream, and can be incorporated into plant proteins and polysaccharides for making meat alternatives, plant-based cheese, and pastry (Nikiforidis et al., 2014; Karefyllakis et al., 2019). Aqueous extraction of crushed hydrated oilseeds yields both oleosomes and water-soluble proteins in water, which is usually followed by filtration and centrifugation to concentrate oleosomes. The oleosome extraction parameters, including the degree of grinding, oilseed/water ratio, extraction time, and temperature, significantly affect the extraction yield (Rhee et al., 1972). After centrifugation, up to 75%–90% (w/w) aqueous suspension of oleosome (cream layer) can be separated (Nikiforidis and Kiosseoglou, 2010; Nikiforidis et al., 2014).

The low chemical and microbial stability of oleosomes is the main barrier to their use in food products (Chang et al., 2013). The chemical stability of the oleosome suspension and its flocculation is mainly dependent on electrostatic attraction and repulsion interactions between oleosome-associated surface proteins (Iwanaga et al., 2008). The charge properties of the oleosomes’ surface can be a major factor contributing to the stability of the oleosomes, and changing pH can have a large effect on their stability (Qi et al., 2017). Previous studies showed that the isoelectric point (pI) of oleosomes ranges from 5.7 to 6.6, with generally a negative surface charge at neutral pH (Maurer et al., 2013; Garcia et al., 2021). If the pH of the oleosome suspension drops below their pI, they start aggregating because of the relatively weak electrostatic repulsion forces operating between the droplets close to the pI (Sukhotu et al., 2014). This phenomenon severely limits their potential applications in foods.

In this study, we attempted to deposit natural surface-active components such as lecithin and polysaccharides on the oleosome surface to create a multilamellar structure surrounding the oil droplet and provide steric and electrostatic barriers against flocculation and enhance mechanical strength. Multi-lamellar “structured” oleosomes, or coated oil bodies, will have enhanced stability and improved mechanical properties. Moreover, the addition of glycerol to decrease the oleosome suspension's water activity and increase their storage stability, as well as to prevent flocculation, will be discussed. The new structured oleosomes could potentially be added to spreads, cream, and processed cheese at acidic pHs, as well as better withstand processing operations such as pasteurization and homogenization.

## 2 Materials and methods

### 2.1 Materials

In our study, sunflower oleosome suspensions in water (70% oil content w/w), with 0.4% antimicrobial preservative (Neolone) and without preservative, were supplied by Botaneco (Calgary, Alberta, Canada). The de-oiled sunflower lecithin powder (Sunlec 25) was purchased from Perimondo (New York, USA), and polysaccharides (carrageenan, pectin, fenugreek, locust bean, guar, gellan, and xanthan) were received as a gift from Caldic (Mississauga, Ontario, Canada). The food-grade glycerol was purchased from Ingredient Depot (Brossard, Quebec, Canada). The fluorescent dyes, including Nile blue, and Rhodamine B, were purchased from Sigma-Aldrich (Oakville, Ontario, Canada). The composition of the sunflower lecithin, both phospholipid species and fatty acid composition is given in Hanley et al. (2024).

### 2.2 Coating oleosomes with lecithin, polysaccharides, and glycerol addition

To understand the effects of adding glycerol, lecithin, and polysaccharides on increasing the physical stability of oleosomes, a mixture of 40% (w/w) glycerol, 40% (w/w) oleosome stock, and 20% (w/w) buffer (25 mM NaHCO_3_) was mixed and homogenized at 15,000 RPM (IKA magic LAB, Wilmington, NC, USA) for 1 min. In comparison, coated oleosomes with lecithin were prepared by adding 0.05%, 0.1%, 2%, 3%, and 10% (w/w) lecithin to a 10% dilution of the oleosome stock in water, using a magnetic stirrer for an hour at room temperature (22 °C), followed by homogenization at 15,000 RPM for 1 min. To study the effects of coating oleosomes with hydrocolloid gums on increasing their colloidal stability, we first tested the solubility of 0.1% (w/w) gums (carrageenan, pectin, fenugreek, locust bean, guar, gellan, and xanthan) in water. Pectin, carrageenan, and xanthan showed high solubility in water at room temperature, while the solubility of Fenugreek, guar, and gellan gums in water was quite low. Heating above room temperature and mixing were necessary to solubilize fenugreek, guar, and gellan gums in water. Gellan was solubilized in water only after 30 min of heating at 90 °C.

In the second step, after completely dissolving gums in water, the pH of 0.1% (w/w) gum solutions in water was decreased to 4.0 using 0.1M HCl. Then oleosomes were added to each gum solution and mixed for 30 min to obtain final concentrations of 10% (w/w) oleosomes in the gum dispersions, followed by homogenization at 15,000 RPM for 1 min.

### 2.3 Particle size analysis

The particle size distribution of the oleosomes was evaluated using a static multi-angle light scattering (Mastersizer, 2000; Malvern Instruments Co., Ltd., Worcestershire, UK). The oleosome samples were diluted with sodium carbonate buffer solution (25 mM NaHCO_3_) in different concentrations to a pH of 8.3. The relative refractive index of oleosome suspensions and the continuous water phase was 1.474 and 1.330, respectively. Results were reported as the volume surface mean diameter (D[3,2]) and volume-weighted mean diameter (D[4,3]).

### 2.4 ζ-potential analysis

The ζ-potentials of the oleosomes were determined using a ζ-potential analyzer (Malvern Instruments, Malvern, UK). The oleosome suspension (70% w/w) was diluted in water to the concentration of 0.001% (w/v). The pH of the diluted suspension was set in the range of 3.0–9.0 using hydrochloride acid (0.1M) or sodium hydroxide (0.1 M). Then after, the ζ-potential of samples was determined at 25 °C.

### 2.5 Melting point analysis

A differential scanning calorimetry (DSC) model TA Q2000 (TA Instruments, Mississauga, ON, Canada) was used to determine the melting points of oleosome suspension (70% w/w). Nitrogen was used to purge the system at 18 mL/min flow rate. This study determined the melting point of 70% (w/w) oleosome by heating samples from 20 °C to 80 °C at the heating rate of 5 °C min^−1^.

### 2.6 Static surface tension measurements

Static surface tension measurements were conducted at the air-water interface using a Sigma force tensiometer (Biolin Scientific, Linthicum Heights, MD, United States) and a DuNuoy ring. Measurements were conducted immediately after transferring samples. The surface tension provided was estimated using the equation of γ=(F/4πRf). Where F is the maximum force measured when pulling the Du Nuoy ring out of the water phase and overcome surface tension, R is the average radius of the Du Nuoy ring used, and f is the Huh and Mason correction factor calculated as f = R/r (R = radius of the Du Nuoy ring and r = radius of the wire).

### 2.7 Compression isotherms

Compression isotherms of samples were measured at the air-water interface using a Kibron Microtrough G1 Langmuir-Blodgett trough (Kibron, Sweden), controlled using KBN LayerXPro software (Kibron, Sweden). After transferring samples in the trough, interfacial films were immediately compressed from 16,500 mm^2^ to 1650 mm^2^, using mobile barriers moving at a speed of 30 mm/min. The pressure was monitored using a Wilhelmy plate during each compression.

### 2.8 Confocal laser scanning microscopy (CLSM)

The CLSM analysis of oleosomes and their potential aggregation was studied visually using an inverted confocal laser scanning microscope Leica DM IRBE (Leica Microsystems, Heidelberg GmbH, Germany) and a Leica TCS SP2 (Leica Microsystems, Heidelberg GmbH, Germany). After dilution in sodium carbonate buffer solution (5% w/v), the oleosome samples were dispersed on a microscope slide (5 μL). Then the prepared oleosome suspensions were stained (10% v/v) with different dyes (Nile Blue (0.01% w/v, and Rhodamine B (0.05% w/v). After covering the microscope slides with a coverslip, it was fixed using nail polish on the corners of the coverslip. Microscope slides were stored overnight to penetrate the dye in the oleosome structure better. Ar/Kr and He/Ne lasers were operated at excitation wavelengths of Nile Blue, and Rhodamine B at 488 nm, and 510 nm, respectively. The confocal images were recorded at a magnification of 20X and 63X.

### 2.9 Oleosome polysaccharide complex suspension stability test

In this analysis, suspension of oleosome in aqueous solutions containing 0.1% (w/w) of gellan, or 0.1% (w/w) of xanthan that already set their pH at 4.0 using HCL (0.1M) was prepared (final concentration of 10% w/w). After adding oleosomes in the gum solutions, they were mixed for 30 min and the oleosome-polysaccharide mixtures were homogenized by passing through a rotostator homogenizer at 15000 RPM for 1 min. To compare their stability (phase separation as a function of time), the homogenized oleosome-polysaccharide mixtures were stored at 30 °C for 24 h.

### 2.10 Light microscopy

The morphology of oleosomes was studied using an OMAX light microscope (China). The diluted oleosome suspension (10% w/w) was pipetted on a microscope glass slide and coved with a glass cover. The oil bodies were observed with 20X and 40× objective lenses.

### 2.11 Rheological analysis

The rheological properties of the oleosome suspensions were analyzed using both dynamic and static rheological properties. For this purpose, an Anton Paar MCR 302 rheometer (Saint-Laurent, Quebec, Canada) with a cuvette cell geometry (concentric cylinder) was used to test the following parameters.

#### 2.11.1 Amplitude sweep test

In this analysis, the shear strain (oscillating) was a logarithmic profile ramp with initial at 60% and final at 200%. For angular frequency, the constant profile set at 1 S^−1^. The analysis temperature was set at 20 °C.

#### 2.11.2 Frequency sweep test

For the dynamic frequency sweep measurement, the shear strain (oscillating) was constant at 100% value and angular frequency profile was ramp logarithmic, initial at 0.1 S^-1^ and finalized at 200 s^−1^. The analysis temperature was set at 20 °C.

#### 2.11.3 Flow curve analysis

In this analysis, the shear rate was set on a ramp linear profile, starting at 10 S^−1^ to 100 S^−1^. The analysis temperature was set at 20 °C.

### 2.12 The effect of salt (NaCl) on oleosome surface charge

In this experiment, first different concentrations (0 M, 0.01 M, 0.02 M, 0.05 M, and 0.1 M) of sodium chloride in de-ionized (DI) water were prepared. Then a 10% oleosome in DI water was mixed for 10 min and homogenized using a rotostator at 15,000 RPM for 1 min. The final pH of the suspension was 8.14. In the next step, 0.25 ml of 10% oleosome suspension was added to each saline solution and mixed for 1 min. The final pH of saline solutions was 6.2. In the final step, the ζ-potentials of the oleosomes in saline solutions were determined using a ζ-potential analyzer.

### 2.13 Water activity analysis

The water activity analysis was performed using an Aqua Lab water activity analyzer model 4 TEV (Decagon Pullman, WA, USA) with the temperature control. The analysis temperature was set at 21 °C. To calibrate the water activity analyzer, two standard solutions were used including a 6 M sodium chloride in water (aw = 0.76), and a 13.41 M lithium chloride in water (aw = 0.25).

### 2.14 Statistical analysis

All experiments were carried out in triplicate, and the results are presented as average ± standard deviation. Statistical analysis was carried out using GraphPad Prism software version 5.0 (La Jolla, CA, USA). All analyses were run in duplicates and results were stated as mean values ± standard deviations. Data were evaluated using one-way ANOVA and the probability of p < 0.05 was considered significant.

## 3 Results and discussion

### 3.1 Evaluation of oleosome suspension

**Fig. 1:**
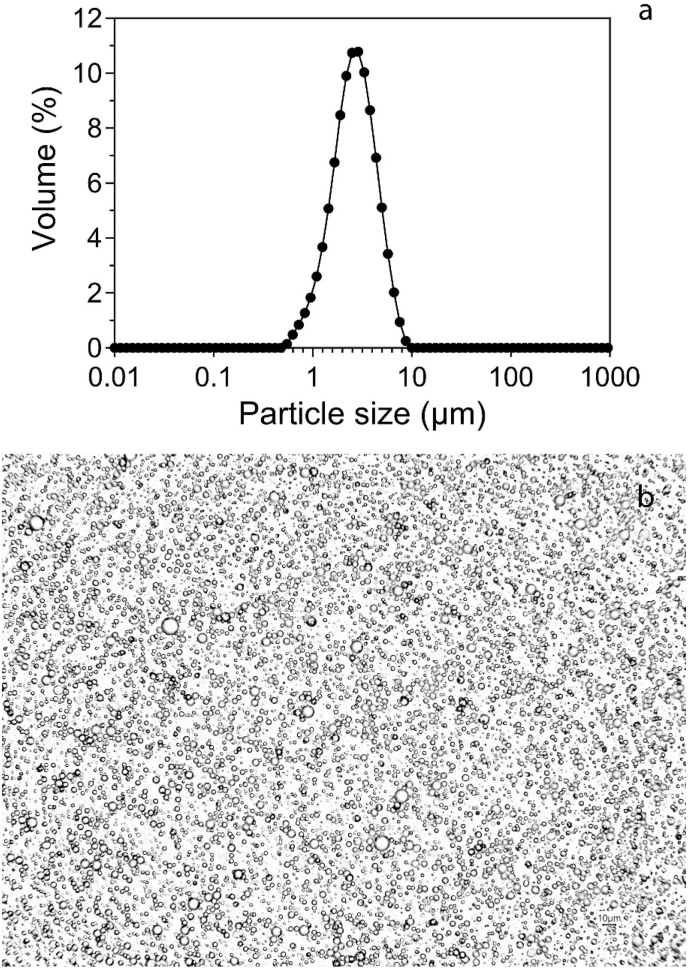
Particle size distribution (a) and optical microscopy image (b) of sunflower oleosome suspension in water used in this study.

In order to determine the initial characteristics of our sunflower olesome suspensions (70% w/w oil), the pH, particle size distribution, ζ-potential, and microstructure were evaluated. The results showed the pH of the commercial oleosome samples was 7.79 ± 0.05, and the oleosomes were homogeneously dispersed in water and no precipitation was observed. The average diameter of the oleosomes was D[4,3] (volume-mean diameter): 5.12 ± 0.44 μm and D[3,2] (Sauter-average diameter): 3.83 ± 0.62 μm. The ζ-potential of oleosomes at pH 7.8 was −42.4 ± 2.1 mV. The particle size distribution and the morphology of oleosomes are shown in Fig. 1. Yang et al. (2023) showed that extracted oleosomes from sunflower seeds had a diameter of up to 10 μm. In another study, Tzen et al. (1993) showed oleosomes extracted from the seeds of rapeseed, mustard, cotton, flax, corn, peanut, and sesame had average diameters that ranged from 0.6 to 2.0 μm.

**Fig. 2:**
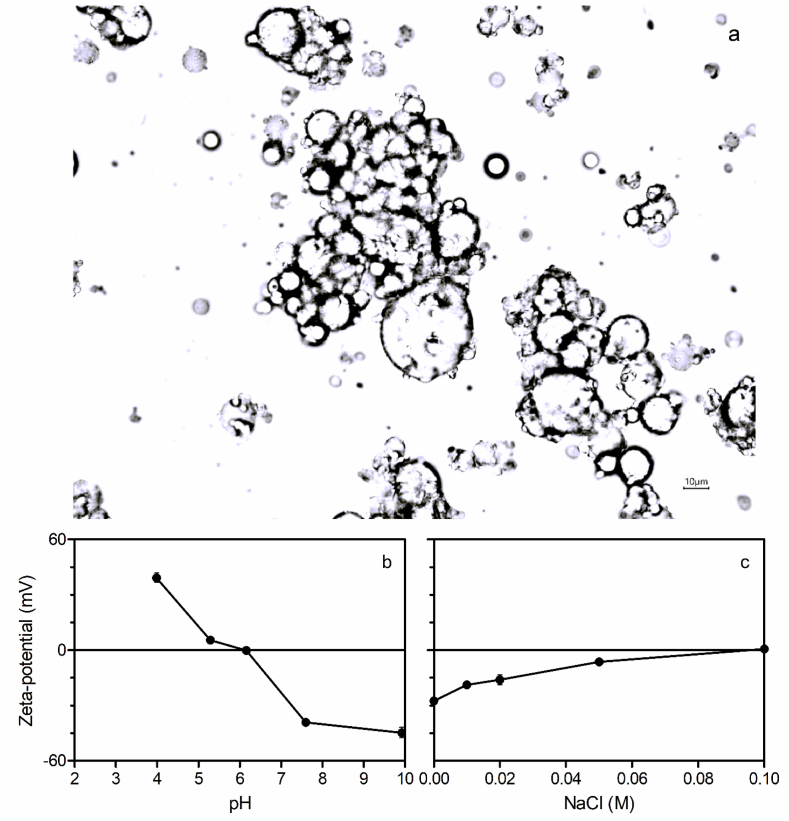
(a) Brightfield light micrograph of a destabilized 10% sunflower oleosome suspension in water at pH 6.5. (b) Changes in ζ-potential as a function of pH of the oleosome suspension. (c) Changes in ζ-potential as a function of ionic strength at pH 8.14 of the oleosome suspension.

To better understand the influence of pH and ionic strength on the colloidal stability of sunflower oleosomes in water, the particle size distribution and ζ-potential were measured in different pH and ionic strengths (Fig. 2b and c).

Fig. 2a shows the flocculation of a 10% (w/w) oleosome suspension in water at pH 6.5. ζ-potential analysis was carried out to determine the surface charge of the oleosomes at different pH values in the range [4–10]. Surface charge is a critical factor affecting the colloidal stability of oleosome suspensions. The ζ-potential analysis showed that the pH stability of oleosomes was very poor, with an isoelectric point of ∼6.2. Fig. 2b shows the change in ζ-potential as a function of pH for sunflower oleosome suspension (10% w/w) in water. The colloidal stability of oleosomes and the rate of flocculation are mainly dependent on the electrostatic attraction and repulsion interactions between oleosome-associated proteins (Iwanaga et al., 2008). The ζ-potential of oleosomes ranged from +40 mV at pH 4.0 to −42.0 mV at pH 10.0, with an isoelectric point of approximately 6.2 (Fig. 2b). So, at lower pH, protonation of the oleosome-associated proteins results in an increase in positive charge, while at high pH, deprotonation takes place, resulting in negatively charged oleosomes. This suggests that oleosomes will destabilize at a pH very close to neutrality and definitely at acidic pHs.

The change in the ζ-potential of oleosomes at different ionic strengths is shown in Fig. 2c. An increase in NaCl concentration resulted in a significant decrease in the ζ-potential of the oleosomes. After the addition of NaCl, the magnitude of the ζ-potential was increased from −30 mV to 0 mV at 100 mM NaCl concentration. In general, increasing the ionic strength of the solution causes a reduction of the charge density of colloidal suspensions due to electrostatic screening effects (McClements, 2016). The negative charge of oleosomes is mainly attributed to surface proteins (mainly oleosins) and phospholipids at the interface, and small changes in the ionic strength of the solution can cause a decrease in the negative charge density of the oleosome interface. Similar results have been reported for the other types of oleosomes, including maize, soybean, and safflower, and a significant reduction in ζ-potential was observed with the addition of salt (Sukhotu et al., 2014; Iwanaga et al., 2007). Yang et al. (2023) showed strong interactions between phospholipids and membrane proteins provide a high stability against coalescence. The aggregation of oleosomes and coalescence that follows at low pH and high salinity will severely limit the application of oleosomes as food ingredients in many food products (Nikiforidis et al., 2014).

### 3.2 Improving the physical stability of oleosomes

#### 3.2.1 Effect of adding glycerol on oleosome flocculation and stability

Addition of glycerol to the oleosome suspension was initially carried out to improve on the microbial stability of the system. Oleosomes are not very stable from a microbiological perspective and Neolone is usually added as a biocide at 0.4% (w/v) concentration. It is used in the cosmetics and personal care industry and is a blend of methylisothiazolinone and phenoxyethanol. However, it is not allowed in foods. We added glycerol to reduce the water activity of the system and enhance microbial stability.

However, we also noticed some structural changes. Homogenization of oleosomes with glycerol caused a significant reduction in the average diameter of oleosomes from 5.12 ± 0.44 μm to 2.63 ± 0.16 μm. This decrease in average size was due to a breakup of oleosome flocs and the prevention of re-flocculation. Here we break up the flocs using a rotostator, which supplies a relatively gentle shear in the presence of glycerol, which prevents the re-flocculation of the oleosomes after the shear has been removed. This deflocculation process can be understood from a decrease in van der Waals' interactions between oleosomes upon glycerol addition (Gögelein et al., 2012; Gräbner et al., 2014). This relies on the Lifshitz approximation of the Hamaker coefficient in van der Waals’ interactions (Acevedo et al., 2011). The dielectric constant of glycerol is much lower than that of water, while it refractive index is higher. When the absolute values of these parameters are introduced into the Lifshitz expression, one can calculate a net decrease in the magnitude of the Hamaker coefficient, which translates of a decrease in attractive interactions between macromolecular structures (Gögelein et al., 2012). The result of this would be a de-flocculation of the oleosomes, which is mediated by van der Waals interactions.

**Fig. 3:**
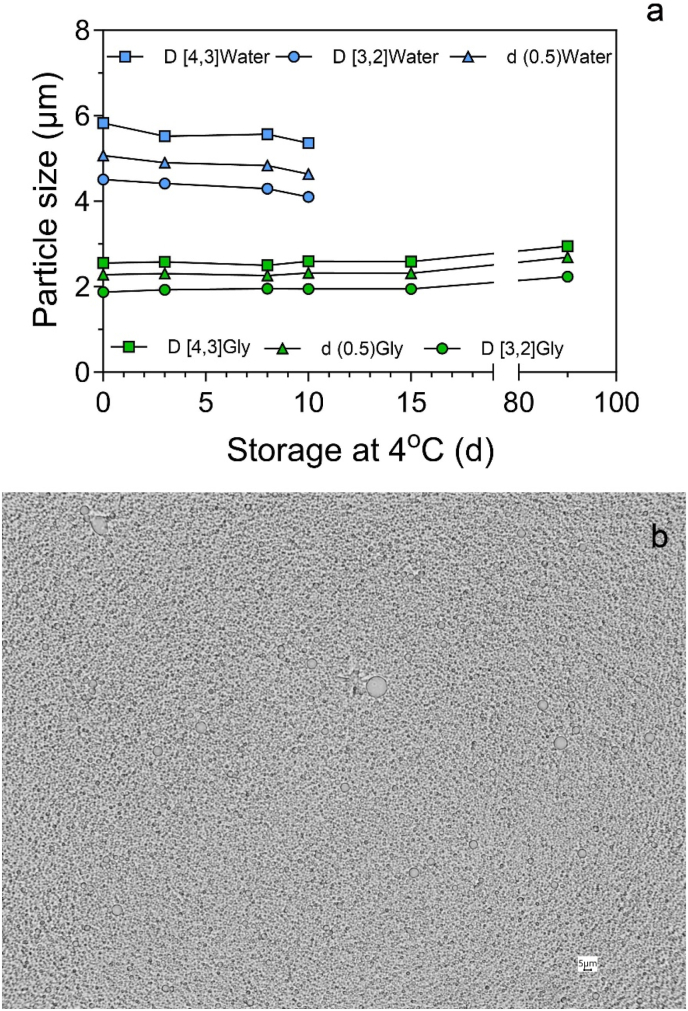
Changes in oleosome size distribution for a homogenized 10% oleosome suspension for a period of 10 days, or diluted with 40% (w/w) glycerol plus 20% carbonate buffer after storage at 4 °C for 3 months. The bottom light micrograph is of oleosomes homogenized and diluted with 40% (w/w) glycerol plus 20% carbonate buffer and stored for 3 months at 4 °C.

Changes in the particle size distribution of the oleosome mixture in the presence (3 months) and absence (10 days) of glycerol at 4 °C are shown in Fig. 3. No significant changes in the average particle size of the oleosomes in glycerol were observed (Fig. 3a and b). Moreover, no mold growth was observed either.

**Fig. 4:**
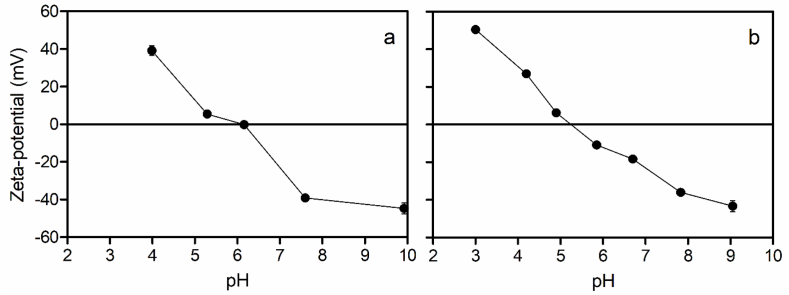
Changes in ζ-potential as a function of pH for (a) a homognized 10% oleosome suspension, and (b) homogenized and diluted with 40% (w/w) glycerol plus 20% carbonate buffer.

The addition of glycerol to oleosome suspensions decreased the isoelectric point of the oleosomes from 6.3 in the absence of glycerol to 5.3 in the presence of glycerol. This was determined from changes in the ζ-potential at different pHs (3–9), as shown in Fig. 4.

The addition of glycerol to oleosome suspension also led to a decrease in the water activity of glycerol/oleosome mixture from 0.993 ± 0.001 to 0.856 ± 0.002, which potentially can limit microbial growth. Glycerol has been extensively used to control water activity in food and pharma (Marcolli and Peter, 2005).

#### 3.2.2 Coating oleosomes with phospholipids

The main purpose of adding phospholipids to the oleosome suspension was to improve the mechanical and chemical stability of the oleosomes. The effect of the addition of 0.2% (w/w) lecithin on oleosome particle size is shown in Table 1.

**Table 1:** Measured size (μm) of sunflower oleosomes upon deposition of 0.2% (w/w) sunflower lecithin on their surface.

Sample	D [4,3]	D [3,2]	d (0.5)
**10% oleosome (rep. 1)**	4.577 ± 0.003	3.380 ± 0.003	4.105 ± 0.005
**10% oleosome** + **0.2% lecithin (rep. 1)**	4.922 ± 0.016	3.747 ± 0.012	4.437 ± 0.015
**Difference in oleosome diameter (μm)**	0.345 ± 0.013	0.367 ± 0.009	0.332 ± 0.01
**10% oleosome (rep. 2)**	5.114 ± 0.377	3.559 ± 0.241	4.421 ± 0.315
**10% oleosome** + **0.2% lecithin (rep. 2)**	5.561 ± 0.325	3.864 ± 0.137	4.819 ± 0.284
**Difference in oleosome diameter (μm)**	0.447 ± 0.052	0.305 ± 0.104	0.398 ± 0.031

**Fig. 5:**
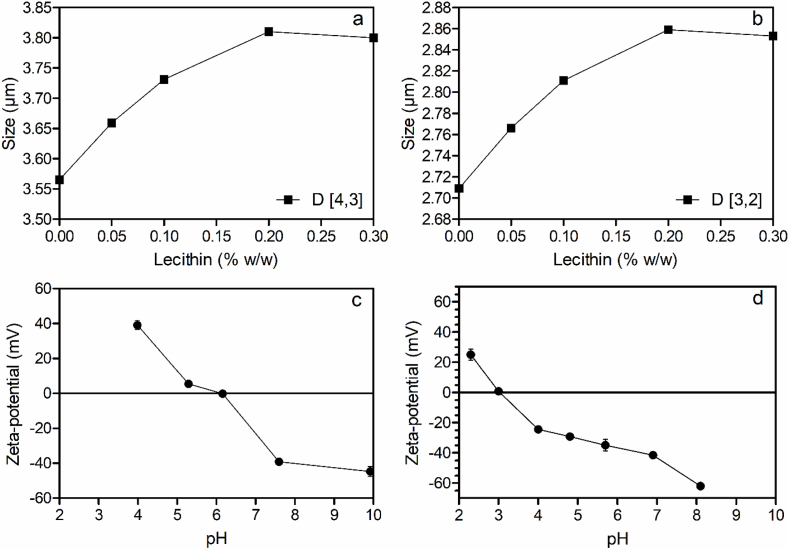
Changes in (a) D[4,3] and (b) D[3,2] of 10% oleosomes exposed to 0.2% (w/w) lecithin and its effect on the ζ-potential as a function of pH (c) before and (d) after coating.

Statistical analysis (unpaired *t*-test with Welch's correction) of the mean differences in particle size distribution showed a significant increase in the diameter of 10% oleosomes coated with 0.2% lecithin. For example, based on increases in the D [4,3] value in Table 1, we estimate a 447 nm deposition on the oleosome surface. We have thus effectively created a “coated” oleosome with properties that significantly differ from the original one. We proceeded then to generate binding curves (Fig. 5). Based on the obtained results, it is clear that the addition of lecithin to oleosomes lead to an increase in oleosome size. This increase in diameter is not due to an artifact in the size distribution profile of the oleosomes with added lecithin, since all distributions were monomodal and narrow (Supplementary Information).

The deposition of lecithin on the oleosome surface caused a shift in the pI to lower pH values. Fig. 5c shows the ζ-potential vs. pH profile for coated oleosomes with 1% lecithin. The ζ-potential of liposomes alone is between −45 and −55 mV in the pH range studied (Figure S2). This suggests that coated, or structured, oleosomes will withstand much better acidic conditions. Therefore, their colloidal stability is enhanced, and potentially their usability in food products can be improved.

**Fig. 6:**
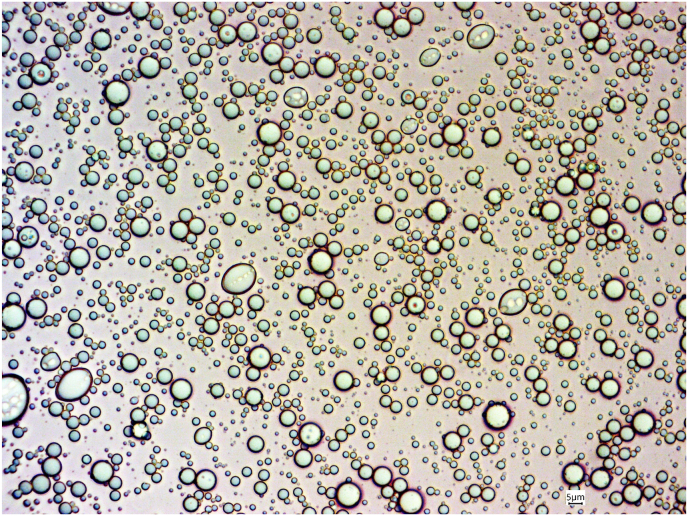
Light micrograph of Rhodamine B-stained 10% oleosomes, the red surface staining shows the sunflower lecithin-coated oleosomes. (For interpretation of the references to colour in this figure legend, the reader is referred to the Web version of this article.)

Since rhodamine B forms adducts with phospholipids and does not require a heat treatment, it was used to stain lecithin-coated oleosomes (Castro et al., 2012). The light micrograph image showed a more prominent surface staining of the lecithin-coated oleosomes (dark-red circles), corroborating the finding that oleosomes are coated with the sunflower lecithin (Fig. 6).

In order to compare the physical stability between coated oleosomes with lecithin and oleosomes without lecithin at low pH in the presence of shear, a 50% oleosome sample was mixed with 1% (w/w) sunflower lecithin at pH 5.0 and homogenized at 15,000 RPM for 1 min using a rotostator. The samples were then stored at 4 °C for five days. A greater physical stability of lecithin-coated oleosomes relatvive to uncoated homogenized oleosomes was observed macroscopically (Figure S1).

**Fig. 7:**
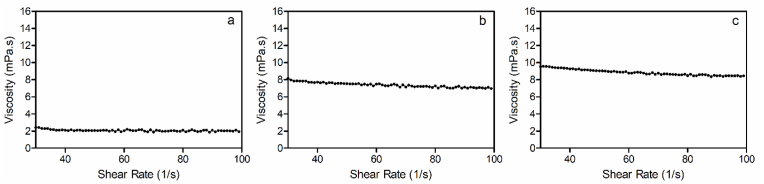
Flow curves for (a) 1% liposomes, (b) 50% uncoated oleosomes, and (c) 50% oleosomes coated with 1% lecithin.

In the next part of this study, the rheological characteristics of coated oleosomes with lecithin were compared to uncoated oleosomes. For this purpose, a traditional flow curve (viscosity vs. shear rate) of samples showed an unstructured liquid with a classic Newtonian behavior, and a constant viscosity as a function of shear rate (Fig. 7). Upon addition of 1% lecithin to a 50% oleosome suspension, the viscosity of the oleosomes increased form 6.9 mPa s to 9.4 mPa s at 40s^−1^. This suggests a larger particle size or some oleosome-oleosome interactions. Interestingly, the viscosity of the liposomes was only 2.15 mPa s. Thus, the observed effect is clearly due to the coating of the oleosomes. The average viscosity of samples at different shear rates is shown in Table 2.

**Table 2:** Dynamic viscosity of liposome and oleosomes suspensions.

Sample	Viscosity (mPa.s)
**1% liposomes**	2.16 ± 0.42
**50% oleosomes coated with 1% lecithin**	9.17 ± 0.74
**50% uncoated oleosomes**	7.54 ± 0.48

**Fig. 8:**
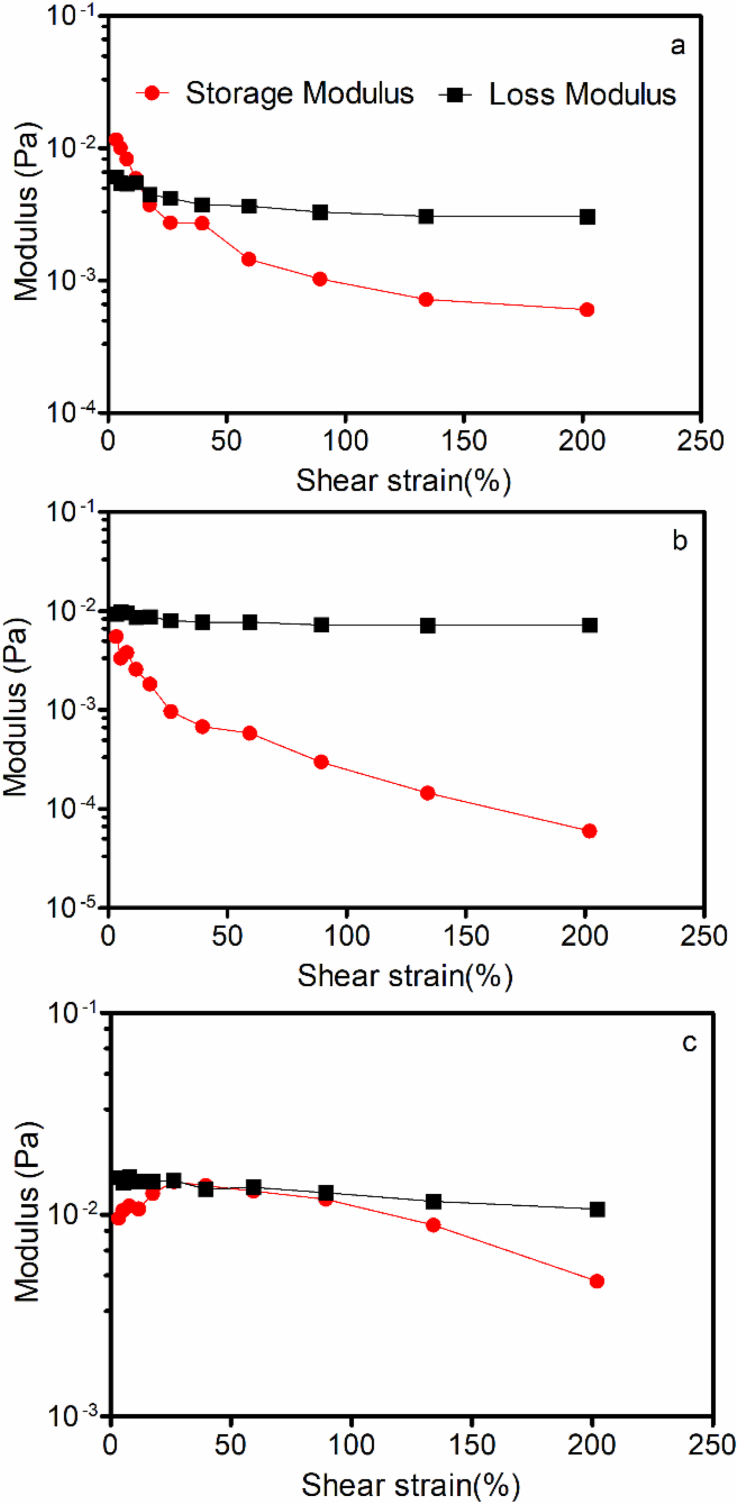
Amplitude sweeps (% shear strain) at a frequency of 1Hz for (a) 1% lecithin liposomes, (b) 50% uncoated oleosomes, and (c) 50% oleosomes coated with 1% lecithin.

**Fig. 9:**
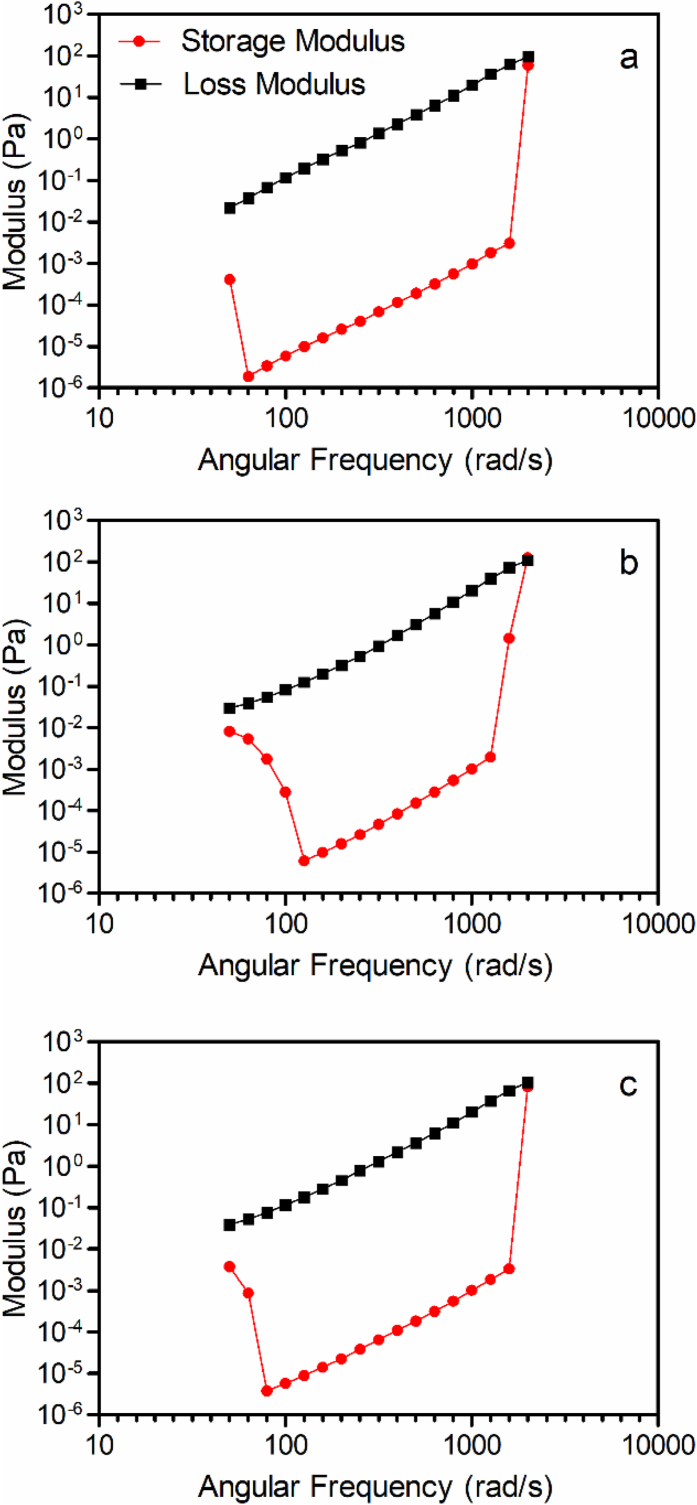
Frequency sweeps (rad/s) at a shear strain of 100% for (a) 1% lecithin liposomes, (b) 50% uncoated oleosomes, and (c) 50% oleosomes coated with 1% lecithin.

The results of dynamic oscillatory rheological measurements, namely as an amplitude sweep and a frequency sweep, are shown in Fig. 8, Fig. 9, respectively. The amplitude sweeps (Fig. 8) clearly show the fact that the material has very weak structure, and the loss and storage moduli are very small. Moreover, the G”, or loss modulus, is greater than the storage modulus (G’), clearly suggesting coated and uncoated oleasomes are, in fact, liquids with very little to no elastic character.

In Fig. 9, frequency sweeps of samples show a strong frequency dependence for both moduli in all samples, and the loss modulus was always greater than the storage modulus. This is characteristic of fluids with no particular structure. The power-law exponent for all samples in the frequency sweep was ∼1.25.

#### 3.2.3 Coating of oleosomes with polysaccharides

Previous studies showed attractive electrostatic interactions formed by protein-hydrocolloids complex interactions could potentially be used to improve the physical stability of oil-in-water emulsions (Zambrano and Vilgis, 2023; Moreau et al., 2003; Guzey and McClements, 2007; Mert and Vilgis, 2021).

Addition of oleosomes to gum dispersions at pH 4.0, increased the pH to different extents depending on the gum. (Table 3). The final pH for 10% (w/w) sunflower oleosome suspension in 1% (w/w) pectin was lower than the pI of sunflower oleosomes (6.2), while for fenugreek, gellan, and xanthan, the final pH was close to the pI of the oleosomes. A higher final pH (7.2) was obtained for sunflower oleosome suspensions in 0.1% carrageenan, locust bean, and guar gums. In the final step, the physical stability of 10% oleosomes in 0.1% gum complex suspensions was compared after storing samples at 30 °C for 24 h.

**Table 3:** Change in pH of 0.1% aqueous solutions of gums before setting to pH 4.0 and after addition of oleosomes.

Gum	pH of 0.1% gum in water	Final pH of 0.1% (w/v) gum in water after adjustment to pH 4.0, followed by oleosome addition
**Carrageenan**	9.3	7.2
**Pectin**	3.6	5.4
**Fenugreek**	5.8	6.6
**Locust bean**	5.9	7.2
**Guar**	6.1	7.2
**Gellan**	5.4	6.4
**Xanthan**	6.7	6.7

**Fig. 10:**
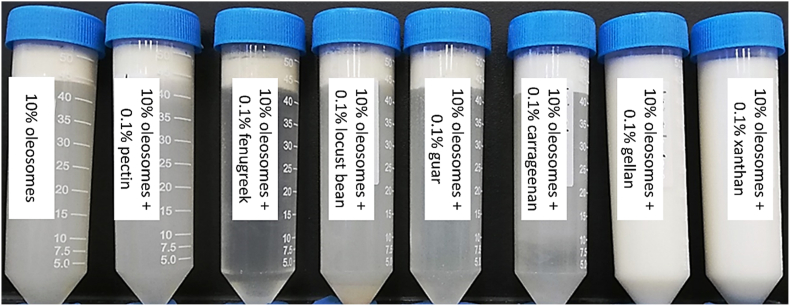
Stability of 10% sunflower oleosomes in 0.1% (w/w) aqueous solution of different gums after lowering the pH, homogenizing, and storing at 30 °C for 24 h.

The results of physical stability are shown in Fig. 10. Colloidal instability was observed for carrageenan, pectin, fenugreek, locust bean, and guar systems, while no phase separation was observed for 10%, sunflower oleosomes coated with 0.1% xanthan or 0.1% gellan hydrocolloids.

**Fig. 11:**
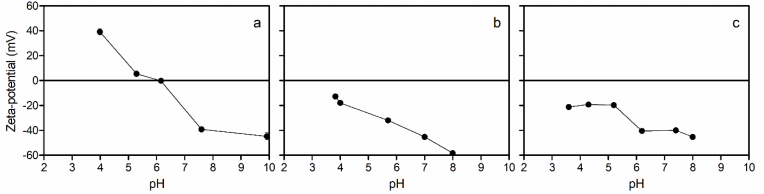
Changes in ζ-potential as a function of pH for (a) 10% native oleosomes, (b) 10% oleosomes suspension in 0.1% gellan and (c) 10% oleosomes suspension in 0.1% xanthan.

Xanthan and gellan are anionic polysaccharides with good temperature, pH, and ionic strength stability. Based on our stability test (Fig. 10), we conclude that xanthan and gellan gums can be adsorbed on the surface of oleosomes at pH values where the oleosomes have a net positive charge on their surface (Gu et al., 2005; Mert and Vilgis, 2021). The addition of 0.1% (w/w) xanthan and gellan gums to 10% oleosomes caused a change in the surface charge of oleosomes. It is generally accepted that the greater the absolute value of the ζ-potential, the stronger the electrostatic repulsive force and the more stable the emulsion. At their pI, oleosomes have poor stability due to aggregation. So, the stability of oleosomes towards aggregation can be improved by coating them with polysaccharides (xanthan and gellan) that form thick charged interfacial layers that increase both electrostatic and steric repulsion between oleosomes. After adding oleosomes to the xanthan and gellan solutions, the pH of the suspension was increased close to the pI of oleosome surface proteins. This could be the reason for the high affinity of xanthan and gellan for the surface proteins on the oleosomes compared to lecithin. In this study, an oleosome complex suspension containing 0.1% xanthan or 0.1% gellan showed a large negative surface charge in the pH range studied, from −60mV at a pH of 8.0 to −13mV at a pH of 3.8 (Fig. 11). Zambrano and Vilgis (2023) showed the ζ-potential of soybean oleosomes changed from −46 mV to + 39.1 mV when the pH of suspension was decreased from 7.0 to 3.0. Since both xanthan and gellan gums are anionic polysaccharides, after addition of 0.1% of gellan and xanthan to 10% oleosome suspension, the ζ-potential showed negative charges in all ranges of pH (Fig. 11b and c).

**Fig. 12:**
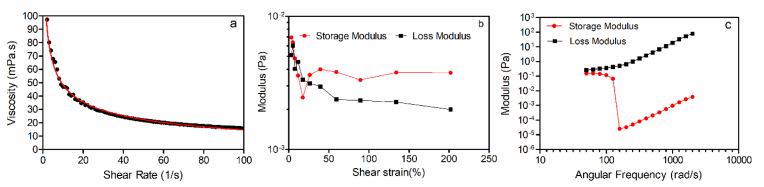
Rheological properties including (a) flow curves, (b) amplitude sweeps and (c) frequency sweeps of 10% oleosomes coated with 0.1% xanthan.

The rheological properties of 10% oleosomes compared to coated oleosomes with 0.1% xanthan were also studied to better understand the effect of adding 0.1% xanthan on viscosity and dynamic shear rheology of the suspensions. Results are shown in Fig. 12. Coated 10% oleosomes with 0.1% xanthan complex suspensions were pseudoplastic (Fig. 12a), and addition of xanthan gum caused an increase in the viscosity of the suspension, which can also affect the stability against separation. The storage modulus (G′) and loss modulus (G″) of 10% oleosome/0.1% xanthan complex suspension were very small, and the amplitude sweep was unremarkable, with the expected G”>G’ observed (Fig. 12b). Kirimlidou et al. (2017) showed interaction of the sesame oleosomes in gelatin (25% w/w) influenced gelation network and resulted in an increase in the storage modulus. The zero-shear viscosity for this sample was 135 ± 1.79 mPa s from a power-law fit to the data. While the viscosity of oleosomes at the shear rate of 10 S^−1^ was 7.74 mPa s, after coating oleosomes with xanthan the viscosity at the same shear rate was increased to 13.16 mPa s.

### 3.3 Static surface tension and compression isotherms of oleosomes

To evaluate the effects of coating 0.12% oleosomes with lecithin and xanthan, oleosomes were mixed with 0.0024% (w/w) lecithin or 0.00024% (w/w) xanthan on static surface tension and compressional behavior, three samples were prepared and were analyzed either immediately or after one day of aging (the pH of the suspension was adjusted to pH = 7.2, using 0.1 M NaOH solution).

**Fig. 13:**
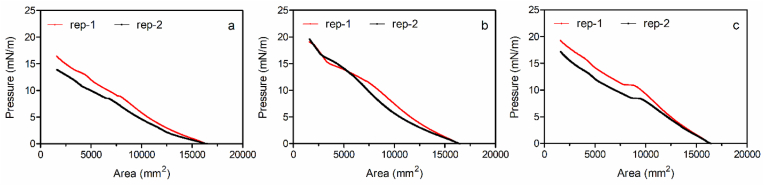
Compression isotherms for (a) oleosomes, (b) oleosomes + lecithin, and (c) oleosomes + xanthan.

These analyses suggest that native, uncoated oleosomes were the most interfacially active (lowest surface tension), and formed the weakest films (lowest maximum surface pressure) upon compression (Table 4). The compression isotherm results are shown in Fig. 13. Addition of either lecithin or xanthan increased film stiffness, as indicated by a higher maximum surface pressure. Moreover, both lecithin and xanthan addition increased the surface tension of the oleosome suspension, indicative of changes in the nature of the oleosome surface and indirectly the surface tension of the suspension. It would seem the surface of the oleosomes had become more hydrophilic upon being coated with xanthan and lecithin.

**Table 4:** Surface tension and maximum pressure upon compression, measured at the air-water interface with oleosome samples.

Sample composition	Surface tension (mN/m)	Maximum pressure upon compression (mN/m)
**0.12% oleosomes**	41.81 ± 0.85^a^	15.2 ± 1.8^a^
**0.12% oleosomes** + **0.0024% lecithin**	45.85 ± 1.00^b^	19.6 ± 0.8^a^
**0.12% oleosomes** + **0.00024% xanthan**	47.25 ± 0.95^b^	18.3 ± 1.5^a^

Different superscript letters within each column indicate significant differences (p < 0.05) between each treatment.

There is the possibility that lecithin displaces adsorbed proteins on the oleosome surface, as reported by Nikiforidis and Kiosseoglou (2011), thus changing both the size and the surface charge of the oleosomes. However, in order for lecithin to displace other interfacially active species, such as proteins, it would have to be more interfacially active than proteins. As a result, the interfacial tension would decrease upon addition of lecithin. We do not observe such a decrease (Table 4), suggesting that lecithin adsorbs at the oleosome surface instead.

## 4 Conclusion

In this study, the interface of oleosomes was engineered to increase their physical stability. De-oiled lecithin and polysaccharides such as xanthan and gellan, as well as plain glycerol addition were studied. Results showed that the coating of oleosomes with lecithin and xanthan plus homogenization effectively decreased particle size, increased thermal stability, and lowered their pI. Moreover, a mixture of 70% oil content oleosomes diluted with 40% glycerol showed a high storage stability at 4 °C, over 3 months. The addition of glycerol prevented flocculation, and also decreased the water activity of the oleosome suspension to 0.85, which potentially could slow down microbial growth.

Lecithin, gums, and glycerol-stabilized oleosomes find extensive applications in the food industry, including but not limited to sauces, salad dressings, coffee whiteners, mayonnaise, alternatives to milk cream, yogurt, and ice cream. Additionally, they can be integrated into plant proteins and polysaccharides to create meat alternatives and plant-based cheeses.

## CRediT authorship contribution statement

**Saeed M. Ghazani:** Conceptualization, Methodology, Formal analysis, Resources, Writing – original draft, Visualization. **Jason Hargreaves:** Conceptualization, Methodology, Supervision, Writing – review & editing, Funding acquisition. **Burcu Guldiken:** Formal analysis. **Analucia Mata:** Formal analysis. **Erica Pensini:** Methodology, Formal analysis, Writing – review & editing. **Alejandro G. Marangoni:** Conceptualization, Methodology, Validation, Supervision, Writing – review & editing, Funding acquisition.

## Declaration of competing interest

The authors declare the following financial interests/personal relationships which may be considered as potential competing interests

Alejandro Marangoni reports financial support was partially provided by the 10.13039/501100000038Natural Sciences and Engineering Research Council of Canada and partially by Botaneco, Inc. One of the co-authors of the paper (Hargreaves) is an employee of Botaneco, Inc.

## Data Availability

Data will be made available on request.
