# Supplementary material for: Oleosome interfacial engineering to enhance their functionality in foods
Source: Curr Res Food Sci. 2024 Jan 17;8:100682. doi: 10.1016/j.crfs.2024.100682 (PMC10831160; doi:10.1016/j.crfs.2024.100682)
Supplement: Multimedia component 1 [file mmc1.docx]

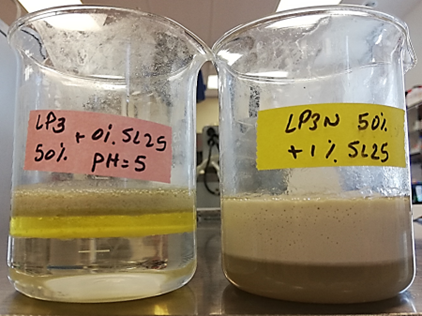


Figure S1. Physical appearance of lecithin-coated (right) vs. uncoated (left) oleosome samples at pH 5.0 after being exposed to high shear conditions and stored at 4 ^o^C for five days.


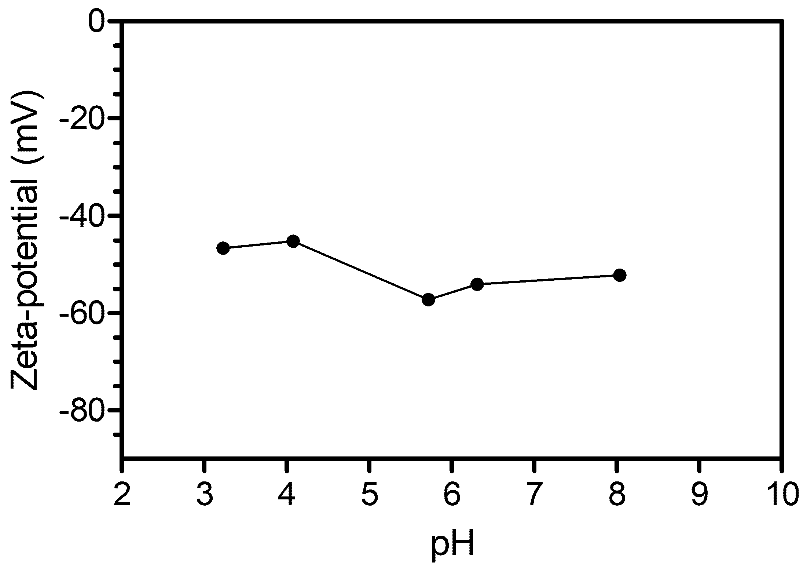


Figure S2. Changes in ζ-potential of 1% liposome suspension in water as a function of pH.
